# The Hungarian version of the oxford cognitive screen (OCS-HU): Normative data and comparison of mild stroke to neurologically healthy adults

**DOI:** 10.1016/j.cccb.2026.100567

**Published:** 2026-10-13

**Authors:** Tímea Tünde Takács, Edina Szabó, Sam Webb, Judit Kárpáti, Károlyné Pálvölgyi, Panna Pálinkás, Orsolya Antal, Júlia Baross, Bernadett Bruckner, Nele Demeyere, Bence Gunda

**Affiliations:** a Semmelweis University, Department of Neurology, Budapest, Hungary; b Semmelweis University, Faculty of Health Sciences, Department of Voice, Speech and Swallowing Therapy, Budapest, Hungary; c Semmelweis University, Faculty of Medicine, Budapest, Hungary; d Bajcsy-Zsilinszky Hospital and Clinic, Department of Musculoskeletal Rehabilitation, Budapest, Hungary; e Doctoral School of Psychology, ELTE Eötvös Loránd University, Budapest, Hungary; f Institute of Psychology, ELTE Eötvös Loránd University, Budapest, Hungary; g Department of Neurology, Bajcsy-Zsilinszky Hospital, Budapest, Hungary; h Nuffield Department of Clinical Neurosciences, University of Oxford, Oxford, United Kingdom

**Keywords:** Post-stroke cognitive impairment, Cognitive screening, Oxford cognitive screen, Stroke rehabilitation, Implementation science, Acute stroke pathways, Neuropsychological assessment, Acute stroke units

## Abstract

**Background:**

Post-stroke cognitive impairment drives long-term disability, yet cognitive deficits are rarely systematically screened in clinical practice or trials. The Oxford Cognitive Screen (OCS) is an established, brief, stroke-specific bedside tool. We present the Hungarian version (OCS-HU) to establish normative data and characterise performance in acute stroke survivors, to enable routine screening.

**Methods:**

In a prospective, multicenter cross-sectional study, 106 healthy controls and 124 acute stroke survivors completed the OCS-HU within three weeks post-stroke. Normative 5th/95th percentile cut-offs derived from controls were compared with UK normative data. OCS-HU performance was subsequently compared between Hungarian controls and acute stroke survivors.

**Results:**

The stroke cohort (68.21 ± 12.41 years) presented with a predominantly mild clinical profile (mean NIHSS: 5.15 ± 3.96; mRS: 2.47 ± 1.49). Hungarian controls (59.61 ± 12.30 years) were younger (p = 0.013) and had lower educational attainment (p < 0.001). Controlling for demographic differences, subtests demonstrated comparable normative performance, allowing retention of original UK cut-offs across most domains with minor linguistic adaptations. Minor control performance differences reflected demographic variations in age and education rather than recruitment bias. Stroke patients performed significantly worse than controls on 10 of 14 assessed measures (p < 0.001). Nonsignificant differences in the broken hearts test, semantics, and praxis reflect the mild cohortseverity and control error variance.

**Conclusions:**

We establishes local normative reference data and characterised OCS-HU performance in acute stroke survivors, providing a foundation for its clinical and research application to improve deficit recognition, highlight unmet rehabilitation needs, and complement standard functional outcome measures.

## Highlights

• Hungarian normative data were established for the Hungarian Oxford Cognitive Screen.
• We establish Hungarian normative data and clinically relevant cut-off scores.
• The original OCS cut-off scores remained comparable across most cognitive domains.
• The OCS-HU supports routine post-stroke cognitive screening and rehabilitation planning.

## Introduction

Acute stroke is a leading cause of disability worldwide [1]. Governments, stroke societies and civil organizations implement media campaigns targeting public awareness and timely ambulance calls, with the aim of increasing the number of eligible patients for acute reperfusion therapies. Alongside these public health efforts, research aims to expand treatment time windows to enhance treatment rates and to lower disability and death measured by the modified Rankin Scale (mRS). Even though the rate of post-stroke cognitive impairment (PSCI) can be as high as 83% [2], most clinical trials do not have an outcome measure targeting cognitive status [[3], [4], [5], [6], [7]]. This gap may reflect the inherent challenges of assessing premorbid cognitive function, as well as the absence of a widely accepted, standardised cognitive screening tool tailored to the stroke population [8].

The Oxford Cognitive Screen (OCS) was developed to address this need. It is a stroke-specific cognitive screening instrument that is freely available for clinical use, can be administered by any trained healthcare professional at the bedside, and is typically completed within 25 min [9]. Its linguistic adaptations are growing in number, broadening its international applicability. Importantly, however, the published language adaptations have followed different evaluation pathways: while some have undergone additional psychometric validation, others have primarily focused on standardised linguistic and cultural adaptation followed by the establishment of population-specific normative data and reference cut-offs [[10], [11]]. Recognizing its potential as the standard tool for PSCI screening, we undertook the linguistic adaptation of the OCS into Hungarian (OCS-HU). The translation methodology and pilot testing have been described previously [12]. The present paper focuses on normative standardisation and clinical benchmarking. Here, we aim to: (1) establish Hungarian normative reference data and population-specific cut-offs, (2) compare local performance against original UK benchmarks, and (3) evaluate known-groups discrimination between healthy controls and acute stroke survivors. Ultimately, this may lay the groundwork for incorporating a stroke-specific neuropsychological screening tool into routine clinical practice in Hungary. Such implementation has the potential to improve the recognition of post-stroke cognitive impairment, identify unmet rehabilitation needs, and inform healthcare resource planning and policy.

## Methods

### Study design

The linguistic adaptation and pilot study of the OCS-HU were conducted following approval by Oxford University Innovation Limited (license number 00OCS-1005,800), in strict accordance with the *Translation and Linguistic Validation Process* and the *Concept Elaboration Report* provided by the developers; the methodology has been described in detail in our previous publication [12]. Briefly, the process involved: (1) forward translation of the original UK version into Hungarian by independent native speakers, (2) reconciliation and synthesis into a primary draft, (3) back-translation into English by an independent translator blind to the original instrument, (4) expert panel review to resolve semantic and conceptual discrepancies, and (5) a pilot feasibility study to assess item clarity, bedside usability, and time requirements prior to normative testing.

The present study addresses the subsequent normative phase by examining OCS-HU performance in a larger Hungarian control sample and in an independent cohort of acute stroke survivors. This is a cross-sectional observational study to determine Hungarian normative data-based cut-off scores for impairment on the OCS. The study was not designed as a comprehensive psychometric validation study of the Hungarian version of the original OCS instrument.

This prospective, multicenter study was conducted at the Department of Neurology, Semmelweis University, and the Neurology and Rehabilitation Departments of Bajcsy-Zsilinszky Hospital and its Outpatient Clinic, Budapest from 15th of December 2024 to 15th of December 2025. Both centers are primary stroke centers with on site thrombolysis and off site thrombectomy offered at Semmelweis University, Department of Neurosurgery and Neurointervention, located 7–10 km away. Since 29th of May 2024, an institutional procedure at Semmelweis University has been in effect mandating that the National Ambulance Service transport all patients with suspected large vessel occlusion - defined by a Rapid Arterial Occlusion Evaluation (RACE) score ≥ 5 [13] - directly to the comprehensive stroke center at the Department of Neurosurgery and Neurointervention, Semmelweis University. Because of this policy, severe stroke patients were less likely to present at our hospitals, leading to a mild stroke cohort in this study.

### Participants

Stroke sample: Consecutive patients aged ≥18 years with acute stroke - defined by ischaemia or haemorrhage on neuroimaging - were prospectively enrolled within three weeks of symptom onset, provided they were able to cooperate and did not meet exclusion criteria. A total of 124 patients were included. Exclusion criteria were impaired consciousness (not alert on the AVPU scale), severe attentional deficits (inability to engage with the assessment), severe visual impairment, inability to sustain cooperation for at least 15 min, or inability to complete the first two OCS tasks due to severe receptive aphasia.

Control sample: A total of 106 control participants aged ≥18 years were recruited, attampted to match as closely as possible to the stroke group for age, sex, and educational level. Participation was voluntary and uncompensated. The control group comprised healthy individuals recruited from visitors with no history of illness affecting cognition, in alignment with the ethics approval. Participant medical histories were obtained directly from the individual and, where available, supplemented by relatives. Alongside healthy individuals, neurology inpatients presenting with conditions that do not affect cognitive function were also included. Inpatients had no known intracranial lesions on imaging and no history of disease affecting cognitive abilities (for example, patients with Parkinson's disease were excluded, while patients with lumbar discopathy were included). Eligibility for inpatients was determined by a neurologist based on available medical history, clinical assessment, and review of relevant imaging and medical information. Minimum age was 25 years, maximum age was 89 years, the age distribution of the control group comprised 1 participant aged 20–29, 4 aged 30–39, 15 aged 40–49, 34 aged 50–59, 24 aged 60–69, 20 aged 70–79, and 8 aged 80–89 years. Participants did not undergo cognitive screening prior to OCS administration, as the OCS was intended to characterise the overall cognitive profile of a Hungarian control sample rather than serve as a confirmatory assessment following pre-screening.

Sample size was determined in accordance with recommendations from the original OCS developers which standardise normative data collection on the basis of sample representativeness and resource use in translations, and not statistical power. Informed consent was obtained from both stroke survivors and controls.

### Procedure

The team of trained healthcare professionals, who were involved in data collation comprisedof a neurologist, a linguist, two speech therapists, a chief nurse, a medical student and a neuropsychologist trainee. OCS Version A or B was counter balanced by the person performing the assessment. The OCS-HU was administered at the bedside or in an outpatient setting by a team member, requiring approximately 15–30 min for the stroke group and 15–20 min for controls. The study was approved by the Ethics Committee of Medical Research Council (approval number: BM/29,860–1/2024).

### Statistical analysis

Demographic characteristics were summarized descriptively. Normative data were derived from the healthy control sample; 5th and 95th percentile cut-offs were calculated for each OCS subtest. We split the data into approximately equal age groups to stratify cut-offs by age in-line with the original UK OCS. Age group selected based upon tertiles and balance of subsample numbers. Hungarian and UK normative samples were compared to account for potential differences in cut-off values. Group comparisons of OCS performance and demographic variables were performed using the ranked-based Analysis of Covariance or chi-squared tests, as appropriate. Stroke survivor performance was examined relative to healthy controls both graphically and statistically. Finally, the prevalence of cognitive impairment in the Hungarian stroke sample was determined.

## Results

Seven controls were excluded from analysis due to anomalous trail making test performance (greater than 2SD distance from mean) on the OCS and were not included in further analyses. In Table 1. we present the demographics for the full Hungarian sample.

**Table 1:** Demographic and clinical characteristics of Hungarian participants.

Characteristic	Healthy Controls (n = 99)	Stroke patients (n = 124)	*p* value
Age, years (mean ± SD), range	59.61 ± 12.30, 25–89	68.21 ± 12.41, 11–94	< .001
Education level, n (%)			< .001
Low (Level 1)	6 (6.1)	17 (13.9)	
Medium (Level 2)	48 (49.0)	87 (71.3)	
High (Level 3)	44 (44.9)	18 (14.8)	
Sex (female), n (%)	42 (42.4)	71 (57.3)	0.047
NIH Stroke Scale (mean ± SD)	–	5.15 ± 3.96	–
Modified Rankin Scale (mean ± SD)	–	2.47 ± 1.49	–
Days between Stroke and OCS (mean ± SD)	–	6.18 ± 4.15	–
Stroke side, n (%)	–		–
Left	–	55 (44.4)	
Right	–	47 (37.9)	
Multiterritorial	–	9 (7.3)	
Posterior circulation	–	13 (10.5)	
Stroke typen (%)	–		–
Ischaemic		113 (91.1)	
Haemorrhagic		11 (8.9)	

*Note.* Low education (Level 1) was considered 6–8 years, mid-level (Level 2) between 12 and ​13 years, and high-level (Level 3) ≥16–18 years, discrepency in education levels was due to controls not reporting education in years between these brackets.

### Normative data

We present the normative data from the healthy control samples in comparison to UK controls, including median, and centile scores in Table 2. Our normative sample consisted of adults aged 25–89 years. Based on maintaining approximately equal sample sizes, we divided controls into age bands of 25–54, 55–65, and >65 which resulted in 34 classified in our ‘young’ group, 35 in the ‘middle’ group and 30 in the ‘older’ group. Further classifications were explored based on original OCS age groups, but subsamples were too small, and other classifications unbalanced norms. We present these classifications as guides only. In Table 3 we present Hungarian age stratified normative cut-off data from 99 controls.

**Table 2:** Normative comparison between original UK OCS normative data and Hungarian OCS data including consensus on cut-offs.

OCS subtask	Measure	UK median	UK 5thcentile	UK 95thcentile	HU median	HU 5thcentile	HU 95thcentile	Stat.	Consensus cut-off
Picture naming (max=4)	Overall accuracy	4	3		4	3		F(1)=21.2, p<.001, $\eta_{p}^{2}$= 0.059	-
Semantics (max= 4)	Overall accuracy	3	3		3	3		F(1)=45.68, p<.001, $\eta_{p}^{2}$= 0.12	-
Orientation (max= 4)	Overall accuracy	4	4		4	4		F(1)=35.49, p<.001, $\eta_{P}^{2}$ = 0.095	-
Visual field assessment (max=4)	Overall accuracy	4	4		4	4		F(1)=0.39, p= .531, $\eta_{p}^{2}$= 0.001	-
Sentence reading (max 15 in UK, 10 in Hungary)	Overall accuracy	15	14		10	**9**		F(1)=1550.75, p<.001, $\eta_{p}^{2}$= 0.82	<9
Number writing (max=3)	Overall accuracy	3	3		3	3		F(1)=1.07, p= .423, $\eta_{p}^{2}$= 0.003	-
Calculation (max=4)	Overall accuracy	4	3		4	3		F(1)=8.29, p= .008, $\eta_{p}^{2}$= 0.024	-
Broken hearts test (max=50)	Overall accuracy	48	42		48	**41**		F(1)=0.53, p= .502,	
$\eta_{P}^{2}$= 0.002									
	<41								
	Object Asymmetry (left inattention > 0, right <0)	0	0^a^	0^a^	0	−**2**	**3**	F(1)=1.78, p= .285,	
$\eta_{p}^{2}$= 0.005									
									
	<−1 & >3								
									
	Space Asymmetry (left inattention > 0, right <0)	0	‐2	3^b^	0	−2	**1**	F(1)=4, p = 0.081,	
$\eta_{p}^{2}$= 0.012									
	< −2 & >2								
									
Praxis (imitation) (max=12)	Overall accuracy	11	8		12	8		F(1)=0.57, p= .502, $\eta_{p}^{2}$= 0.002	-
Verbal memory(max= 4)	Verbal memory recall and recognition	4	4		4	**3**		F(1)=37.43, p<.001, $\eta_{p}^{2}$= 0.1	<3
Episodic memory (max= 4)	Episodic recognition	4	4		4	**3**		F(1)=10.37, p= .003, $\eta_{p}^{2}$= = 0.039	<3
Executive function: task switching (optimal= −1)	Executive score accuracy (sum of single tasks minus mixed task)	−1		4	−1		**3**	F(1)=0.69, p= .502, $\eta_{p}^{2}$= 0.003	>3

Note: Comparisons between normative samples were conducted with rank-corrected ANCOVA. P-values were corrected with Bonferroni-holm corrections. HU refers to Hungary. Values in bold face represent scores differing between UK and Hungarian samples.

**Table 3:** Normative data, descriptives for differing age groups from neurologically healthy control subgroups.

		<54						55–65						>65					
OCS subtask	Subtask scoring	N	Med	Min	Max	5th	95th	N	Med	Min	Max	5th	95th	N	Med	Min	Max	5th	95th
Picture naming	Overall accuracy	34	4	3	4	3		35	4	3	4	4		30	4	3	4	3	
Semantics	Overall accuracy	34	3	3	3	3		35	3	3	3	3		30	3	3	3	3	
Orientation	Overall accuracy	34	4	3	4	4		35	4	4	4	4		30	4	3	4	3	
Visual field	Overall accuracy	34	4	4	4	4		35	4	4	4	4		30	4	2	4	4	
Sentence reading	Overall accuracy	34	10	9	10	**10**		35	10	3	10	**9**		30	10	8	10	**9**	
Number writing	Overall accuracy	34	3	2	3	3		35	3	3	4	3		30	3	2	3	3	
Calculation	Overall accuracy	34	4	0	4	3		35	4	3	4	3		30	4	3	4	3	
Broken hearts	Overall accuracy	34	48.5	41	50	**43**		35	48	40	50	43		30	47	36	50	42	
Broken hearts	Space Asym (left inattention > 0, right <0)	34	0	−2	3	**−2**	**1**	35	0	−2	4	**−1**	**2**	30	0	−7	4	**−4**	**4**
Broken hearts	Obj Asym (left inattention > 0, right <0)	34	0	−4	2	**−1**	**1**	35	0	−2	1	**−1**	**0**	30	0	−3	3	**−3**	**2**
Imitation	Overall accuracy	34	12	8	12	**9**		35	12	8	12	8		30	12	7	12	8	
Recall and recognition	Overall accuracy	34	4	3	4	**3**		35	4	3	4	3		30	4	1	4	1	
Episodic Memory	Overall accuracy	34	4	3	4	**3**		35	4	3	4	3		30	4	2	4	3	
Executive task	Exec score acc (	34	−1	−7	5	−2	**0**	35	−1	−4	3		**3**	30	−1	−4	5		3

Note. Values in bold face represent scores differing between UK and Hungarian samples.

When comparing Hungarian and UK normative samples, we found significant differences in education level (UK median education level = 3, Hungarian median education level = 2, x2(2) = 63.10, p<.001) and in age of sample (UK mean age = 63.88 (SD=13.10) Hungarian mean age = 59.61(SD=12.50), t(207.32)= 2.50, p = 0.013), but not in distribution of sex where most were female (x2(1) = 0.02, p>.05) [4]. Test version A was performed by 73 stroke patients and 46 controls, and version B by 53 controls and 51 stroke survivors.

No differences were found for visual field, number writing, broken hearts test (whole), praxis, nor executive function between control groups, and differences did not materially change cut-offs. That is, for sub scores measured on the same scale (not sentence reading), cut-offs were vastly similar between groups (4 exceptions out of 16). Differences in cut scores existed for the broken hearts test (accuracy and sub scores), verbal recall, and executive function. Differences were not extreme. The difference between UK and Hungarian broken hearts accuracy was unsubstantial, but differences in neglect sub scores were substantial so new cut-offs were used. Overall, cut-offs remained similar to the original OCS, except where cultural differences did not permit similar scores (sentence reading) or small differences remained in sample. Whilst cut-offs remained same, the underlying samples were different from each other.

### Stroke sample performance

We then compared the Hungarian (HU) stroke sample to the healthy HU control sample on their performance on the OCS. Eighty-three percent of stroke patients were assessed at Semmelweis University, Department of Neurology. Fig. 1. shows a graphical representation of sample overlap, visualised with boxplots overlayed with visually jittered data points to show data distributions.

**Fig. 1:**
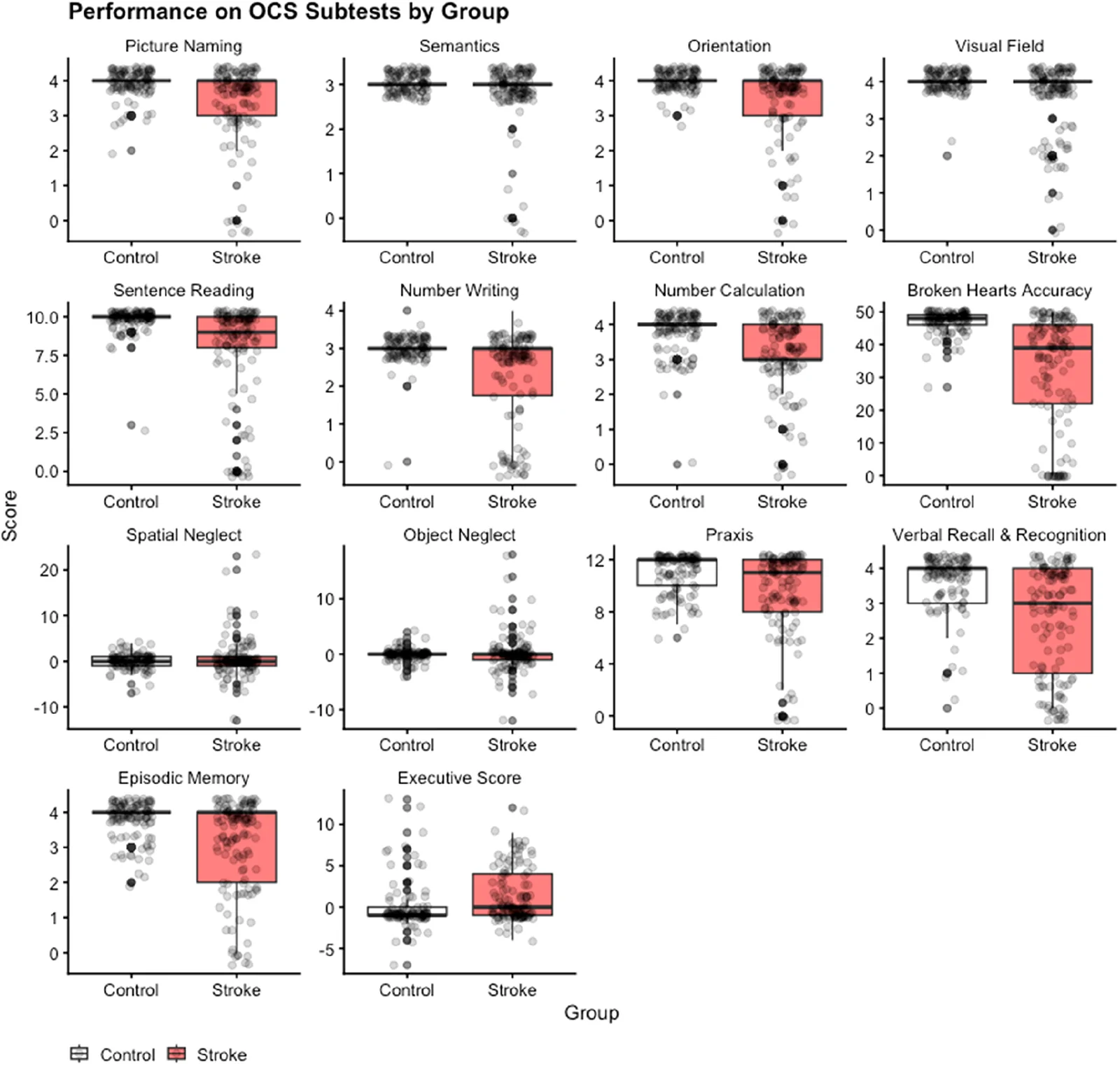
Boxplot performance of Hungarian stroke and healthy control samples on OCS subtests, overlaid with visually jittered data points to enable better data visualisation.

A series of rank-based analyses of covariance (ANCOVAs) controlling for age and education revealed statistically significant main effects of sample group across most Oxford Cognitive Screen (OCS) subtests, including Picture Naming, F(1, 223) = 9.49, p = .002, Orientation, F(1, 223) = 7.79, p = .006, Visual Field, F(1, 223) = 11.31, p = .001, Number Writing, F(1, 223) = 19.49, p < .001, Number Calculation, F(1, 223) = 12.51, p < .001, Sentence Reading, F(1, 223) = 21.93, p < .001, Broken Hearts Accuracy, F(1, 223) = 33.56, p < .001, Verbal Recall & Recognition, F(1, 222) = 13.28, p < .001, Episodic Memory, F(1, 223) = 7.00, p = .009, and Executive Score, F(1, 223) = 5.26, p = .023; whereby controls performed better than stroke survivors. For executive function, the controls (even after exclusion) were diverse in their performance (Fig. 1), with many performing in what is considered ‘impaired’ compared to UK norms.

Conversely, no significant group differences were observed for Praxis, F(1, 223) = 2.30, p = .130, Semantics, F(1, 223) = 2.08, p = .151, Spatial Neglect, F(1, 223) = 0.04, p = .838, nor Object Neglect, F(1, 223) = 0.02, p = .900.

### Proportions of sub-test level cognitive impairment

Finally, we examined proportions of patients with subtest-level impairment in our mild stroke cohort. We calculated the proportion of the stroke sample that scored in the impaired range determined by the newly calculated normative data. See Table 4 for proportions.

**Table 4:** Proportions of stroke participants who scored in the impaired range of scores per OCS subtasks, classified using 5th or 95th centiles from the healthy controls performance.

Task	% Impaired
Picture naming	16.94
Semantics	8.06
Orientation	16.94
Visual field assessment	21.77
Sentence reading	40.32
Number writing	33.87
Calculation	18.55
Broken hearts test	52.42
Space Asymmetry	34.32
Object Asymmetry	23.48
Praxis (imitation)	19.35
Verbal memory recall and recognition	24.39
Episodic recognition	27.42
Executive function: task switching	3.23

## Discussion

In this study, we provide normative data and clinically applicable cut-off values for Hungarian-speaking stroke populations, establishing the Hungarian version of the Oxford Cognitive Screen (OCS-HU). The main finding of this study is that, despite demographic and linguistic differences between the Hungarian and UK samples, the majority of OCS subtests demonstrated comparable normative performance, allowing retention of some of the original OCS cut-off values in most domains. Where cut- offs differed, this was due to difference in range of tests or due to norm group differences. We use the UK normative data as descriptive context for Hungarian OCS norm cut-offs.

### Normative data

When comparing Hungarian and UK normative samples, significant differences were observed in age and educational level. Hungarian controls were younger than their UK counterparts. This difference primarily reflects recruitment challenges, as identifying cognitively healthy older adults without neurological or psychiatric conditions who met all inclusion criteria proved particularly difficult. Similarly, the Hungarian stroke cohort was younger than the UK cohort. This finding aligns with broader population-level health trends, as Hungary has a lower life expectancy and a higher burden of cardiovascular risk factors than the UK and most Western European countries, contributing to an earlier onset of stroke [14]. Furthermore, lower educational levels and socioeconomic disadvantages associated with an increased risk of stroke, may also contribute to these differences [15]. In light of this, we find it acceptable to have a lower age control group for our normative cut-offs and accept statistical comparisons as differences between population level samples rather than a major limitation in the current study. This is also reflected in the education differences between the countries, supported by census data from both nations. In England and Wales, higher education (university degrees or equivalent) is held by 33.8% of adults [16], compared to 21.8% in Hungary, where the population predominantly completes vocational (20.9%) or general secondary schooling (33.4%) [17]. Conversely, lower educational attainment (primary level or no formal qualifications) is higher in Hungary (26.5%) than in the UK (18.2%). Because formal education is a key proxy for cognitive reserve capacity, age and education were considered when interpreting the Hungarian normative data. We acknowledge that our age-stratified normative data represent norms from small subsamples (n=∼35). Whilst the cut-offs were not materially different between UK/Hungarian age groups, providing some validity for their use, we cannot fully interpret all scores using the age norms until larger representative samples are collected*.* Still, these macro-level data confirm that the educational disparities between our Hungarian and UK samples reflect genuine, population-level demographic realities rather than sampling bias. We used age adjusted stratifications for normative data and revealed substantial performance differences for some subtasks (executive score, space neglect, accuracy on broken hearts test), whereby the older the participant the worse they scored, in line with the original UK OCS. These tables offer helpful contextual reference for researchers and clinicians, though standard OCS cut-off scores remain the recommended benchmark for determining cognitive impairment.

### Clinical performance

Among the 14 scored OCS-HU subtests, the majority demonstrated comparable cut-offs between the Hungarian and UK normative samples. Eight subtests showed identical cut-off values, while minor differences were observed in additional subtests, including the Broken Hearts and left and right neglect tasks, memory, and executive function. These differences may be related to the presence of occasional errors among healthy controls, which influenced percentile-based cut-off estimation. We note specifically that the distribution of control scores for the executive functioning subtest were poor, suggesting that compared to the UK sample the Hungarians performed worse and scores in ’impaired’ ranges when looking ath the UK contetx. This is surprising given the Hungarian cohort are younger but not in light of lower education. In this case, as we do not know why the controls were poor on the executive functiong test, we use the Hunagrian normative cut-off and cautiosly interpret patient scores for this subtest. A substantial modification of an OCS-HU cut-offs involved the sentence reading subtest, where the maximum score was adjusted from 15 to 10 to reflect the adapted sentence length. This change was necessary because of the agglutinative nature of the Hungarian language. Reading and retaining a 15-word sentence would have disproportionately increased linguistic complexity and affected the comparability of the reading and memory tasks. The adjustment therefore aimed to preserve the intended cognitive construct assessed by the original OCS. The praxis task showed a notable difference in cut-off values between the Hungarian and UK versions, likely due to the variability in performance within the Hungarian control sample.

Stroke patients performed significantly worse than controls on 10 of the 14 assessed cognitive measures. These between-group differences provide clinical context for the distribution of OCS-HU scores in acute stroke. No significant differences were observed in the broken hearts neglect subscores but were present in the overall accuracy score, likely due to the low number of impairment. No differences in groups were found for the executive function test either, which may partly reflect the mild clinical characteristics of our stroke cohort, as patients with severe stroke were preferentially transferred to a comprehensive neurointerventional centre, and the distribution of our control data.

The observed impairment rates across cognitive domains demonstrate the presence of heterogeneous cognitive deficits even in a relatively mild stroke population. This finding is clinically relevant, as cognitive impairment may remain undetected when stroke outcomes are assessed primarily using functional measures such as the mRS.

## Limitations

This study has several limitations. First, although normative data were obtained from a representative control sample, recruitment of older cognitively healthy participants was challenging, resulting in a younger control sample compared with the UK cohort. Second, our stroke cohort mainly consisted of patients with mild-to-moderate stroke due to local referral pathways for severe stroke, limiting the extent to which our findings can be generalised to patients with more severe neurological impairment. Nevertheless, the ability to demonstrate clear differences between stroke survivors and healthy controls within a relatively mild cohort suggests that the OCS-HU can capture clinically relevant cognitive changes even at lower levels of impairment. As cognitive deficits are expected to be more pronounced following more severe strokes, future studies should include patients across the full spectrum of stroke severity.

## Conclusion

In conclusion, this study provides the normative and clinical performance data necessary to support the use of the Hungarian version of the OCS, within the established framework of the original OCS. Given the high prevalence of post-stroke cognitive impairment and the limited inclusion of cognitive outcomes in routine stroke care and clinical trials, the OCS-HU may support the integration of cognitive assessment alongside traditional functional measures. Future studies should examine the association between acute and long-term cognitive performance and functional recovery (Table 4).

## CRediT authorship contribution statement

**Tímea Tünde Takács:** Writing – review & editing, Writing – original draft, Validation, Supervision, Project administration, Methodology, Investigation, Conceptualization. **Edina Szabó:** Investigation, Data curation. **Sam Webb:** Writing – review & editing, Validation, Methodology, Formal analysis, Data curation, Conceptualization. **Judit Kárpáti:** Methodology. **Károlyné Pálvölgyi:** Investigation. **Panna Pálinkás:** Investigation. **Orsolya Antal:** Investigation. **Júlia Baross:** Investigation. **Bernadett Bruckner:** Investigation. **Nele Demeyere:** Supervision, Conceptualization. **Bence Gunda:** Writing – review & editing, Supervision.

## Declaration of competing interest

The authors have nothing to declare.
